# Susceptibility of Tambaqui (*Colossoma macropomum*) to Nile Tilapia-Derived *Streptococcus agalactiae* and *Francisella orientalis*

**DOI:** 10.3390/microorganisms12122440

**Published:** 2024-11-27

**Authors:** Francisco Yan Tavares Reis, Victória Pontes Rocha, Peter Charrie Janampa-Sarmiento, Ágna Ferreira Santos, Márcia Pimenta Leibowitz, Ronald Kennedy Luz, Felipe Pierezan, Sílvia Umeda Gallani, Guilherme Campos Tavares, Henrique César Pereira Figueiredo

**Affiliations:** 1 Department of Preventive Veterinary Medicine, School of Veterinary Medicine, Federal University of Minas Gerais, Belo Horizonte 31270-901, Minas Gerais, Brazil; yan_reis@hotmail.com (F.Y.T.R.); mvvictoriapr@gmail.com (V.P.R.); peterjs_0126@hotmail.com (P.C.J.-S.); marciapimenta.leibowitz@gmail.com (M.P.L.); gcamposvet@hotmail.com (G.C.T.); 2 Department of Veterinary Clinics and Surgery, School of Veterinary Medicine, Federal University of Minas Gerais, Belo Horizonte 31270-901, Minas Gerais, Brazil; agnaferreira.vet@gmail.com (Á.F.S.); fpierezan@ufl.edu (F.P.); 3 Aquaculture Laboratory, Department of Animal Science, School of Veterinary Medicine, Federal University of Minas Gerais, Belo Horizonte 31270-901, Minas Gerais, Brazil; luzrk@yahoo.com; 4 Postgraduate Program in Aquaculture, Nilton Lins University, Manaus 69058-030, Amazonas, Brazil; silviaugallani@gmail.com

**Keywords:** tambaqui, tilapia, pathogenicity, histopathology, streptococcosis, francisellosis, amazon

## Abstract

Nile tilapia (*Oreochromis niloticus*) and tambaqui (*Colossoma macropomum*) are the two most produced freshwater fishes in Brazil. This study investigated the potential pathogenicity of *Streptococcus agalactiae* and *Francisella orientalis*, previously isolated from diseased Nile tilapia, to tambaqui. Experimental infection trials were conducted in juvenile tambaqui at a dose of approximately 10^7^ CFU fish^−1^, assessing clinical signs, mortality, bacterial recovery, and histopathological changes. Results demonstrated that *S. agalactiae* exhibited high pathogenicity to tambaqui, causing rapid disease progression, high mortality (83.33%) within 48 h post-infection, and severe lesions in multiple organs, under the experimental conditions. In contrast, *F. orientalis* infection did not result in mortality or clinical signs, despite bacterial recovery and granulomatous inflammation observed in the tissues. This study highlights the need to consider the potential impact of these pathogens in tambaqui farming.

## 1. Introduction

*Colossoma macropomum,* commonly known as tambaqui, is a freshwater fish native to the Amazon basin and holds significant economic value in Latin America, particularly in Brazil, Colombia, Peru, Venezuela, and Bolivia [1]. In Brazil, tambaqui is the second most farmed fish, with a production of 113.6 thousand tons in 2023, while Nile tilapia (*Oreochromis niloticus*) ranks first, with a production of 442.1 thousand tons in 2023 [2]. The majority of tambaqui production is directed towards domestic consumption, but approximately 79 tons was exported in 2023, with Peru being the primary importer [3].

Despite its economic significance, limited information is available regarding the bacterial pathogens that commonly affect tambaqui. A monitoring study conducted in 2016 reported bacterial prevalence in tambaqui cultivated at Rio Preto da Eva, Brazil (a large production site of tambaqui). *Aeromonas hydrophila* prevalence was 11.33% during the rainy season and 20.67% during the dry season. *Flavobacterium columnare* and *Streptococcus* sp. had a prevalence of 0.67% each, only during the rainy season [4]. Moreover, species such as *A. hydrophila* [5,6], *Aeromonas veronii* [6], *Aeromonas jandaei, F. columnare* [7], and *Edwardsiella tarda* [8] have recently been reported as significant pathogens in tambaqui. These findings highlight the need for further research to understand the impact of bacterial pathogens on tambaqui farming and to develop effective control measures.

In contrast, bacterial pathogens in tilapia have been extensively diagnosed and researched, with *Streptococcus agalactiae* and *Francisella orientalis* being the most frequently reported in Brazilian tilapia farms, causing substantial economic losses [9,10,11,12]. *S. agalactiae* is a Gram-positive bacterium that causes streptococcosis, a disease that leads to high mortality rates and substantial economic losses in the tilapia industry [13]. Infected tilapia exhibit symptoms such as erratic swimming, exophthalmia, and hemorrhages on the skin and internal organs [14]. Outbreaks of *S. agalactiae* are commonly associated with high water temperatures, typically above 27 °C [15,16]. The rapid spread of *S. agalactiae* in aquaculture systems is facilitated by factors such as high stocking densities, poor water quality, and stress. Understanding the epidemiology, pathogenesis, and control measures of *S. agalactiae* is crucial for developing effective strategies to mitigate its impact on tilapia farming and ensure the sustainability of this vital food source [17].

*F. orientalis*, on the other hand, is an intracellular facultative Gram-negative bacterium that causes francisellosis [18]. This disease is characterized by granulomatous inflammation presented as white nodules in internal organs and skin, also leading to splenomegaly and renomegaly [19]. Francisellosis typically occurs in juveniles and fingerlings of tilapia when water temperature is below 25 °C, hence occurring more frequently during colder seasons of the year in Brazil [20]. The impact of francisellosis on tilapia farming is significant, resulting in high mortality rates and considerable economic losses.

Tambaqui and tilapia are farmed in distinct regions of Brazil. Tambaqui is mainly produced in the north, while tilapia farming is concentrated in the south and southeast [2]. However, aquaculture facilities in the northeast and midwest regions may inadvertently facilitate the transfer of bacterial pathogens between these species. This could occur either through the concurrent farming of both species at the same facility or through proximity to other bodies of water, providing potential routes for pathogen transmission.

Implementing biosecurity protocols to prevent the introduction of pathogenic agents in tambaqui aquaculture is of utmost importance for industry sustainability. Given that tilapia and tambaqui are the two main farmed fish species in Brazil, pathogens that affect tilapia may also pose a great risk to tambaqui. Accordingly, this study was designed to assess the pathogenic potential of *S. agalactiae* and *F. orientalis* strains, previously isolated from diseased Nile tilapia, to tambaqui.

## 2. Materials and Methods

### 2.1. S. agalactiae and F. orientalis Strains

Strains SA95 and FNO12 are well-characterized representatives of *S. agalactiae* and *F. orientalis*, respectively. SA95 was isolated from diseased Nile tilapia produced in Alagoas, Brazil, in 2010. It belongs to the serotype Ib, sequence type 927, had its genome sequenced, proteome characterized, and main spectrum profile established by mass spectrometry [21,22]. Similarly, FNO12 was isolated from diseased Nile tilapia produced in Minas Gerais, Brazil, in 2012. It was used to experimentally infect tilapia maintained at 22 °C, establishing a median lethal dose of 3.89 × 10^2^ CFU [23]. Its genome has also been fully sequenced [23,24]. SA95 and FNO12 were stored in brain heart infusion (BHI) broth (KASVI, Pinhais, Brazil) with 15% glycerol and Mueller–Hinton cation-adjusted broth (Becton, Dickinson and Company, Franklin Lakes, NJ, USA) supplemented with 1% VX (Laborclin, São Paulo, Brazil), 1% glucose, and 15% glycerol, respectively. Both strains were maintained at −80 °C until use.

### 2.2. Fish and Experimental Infections

The pathogenicity of *S. agalactiae* SA95 and *F. orientalis* FNO12 to tambaqui was evaluated through experimental infection. Thirty juvenile tambaqui, with an average body weight of 53 ± 19 g, were acquired from the fish bioterium at the Federal University of Minas Gerais, Brazil. The use of fish in this study was approved by the Ethics Committee on Animal Use of that institution (protocol number 378/2019). Upon arrival at the Laboratory of Aquatic Animal Diseases (AQUAVET) at the Veterinary School of the Federal University of Minas Gerais in Belo Horizonte, Brazil, the fish were acclimated to the new water conditions for 15 days in four glass aquaria, each with a total capacity of 57 L of dechlorinated water. During the acclimation period, half of the water volume was renewed every two days to maintain optimal water quality.

The water temperature was maintained at 28 °C for two aquaria designated for the *S. agalactiae* trial. For the *F. orientalis* trial, the water temperature was gradually lowered by 1 °C/day during the last five days of the acclimation period, reaching 22 °C on the day of infection. This temperature adjustment was designed to mimic the conditions during typical tilapia disease outbreaks, as *S. agalactiae* outbreaks usually occur in warmer seasons, when water temperatures reach 28 °C, while *F. orientalis* outbreaks occur in colder seasons, at 22 °C [9,23,25,26]. Dissolved oxygen was maintained at approximately 6 mg/L by continuous aeration flow. The fish were fed twice daily with commercial feed containing 32% protein (Socil, São Paulo, Brazil) at a rate of 3% of their body weight per day.

During the acclimation period, six fish were euthanized by immersion in a benzocaine solution (Sigma-Aldrich, Saint Louis, MO, USA; 300 mg L^−1^) to ensure they were free from bacterial infections. Brain, kidney, spleen, and liver were aseptically collected, streaked onto tryptic soy agar (TSA) (HiMedia, Mumbai, India), Hsu–Schotts agar [27], and cystine heart agar supplemented with 2% hemoglobin (CHA) (TM Media, Delhi, India), to allow growth of bacteria, such as *Streptococcus* spp., *F. orientalis*, *F. columnaris*, *Aeromonas* spp., or *Edwardsiella* spp. During necropsy, internal organs were also examined for the presence of gross lesions that could indicate any detectable subclinical disease. Subsequently, the agar plates were incubated at 28 °C for 48 h. A negative result, indicated by the absence of bacterial growth, confirmed that the batch of fish was not infected.

For experimental infections, *S. agalactiae* SA95 and *F. orientalis* FNO12 were initially grown in 20 mL BHI broth or 20 mL cation-adjusted Mueller–Hinton broth supplemented with 1% VX and 1% glucose (MHB), respectively, at 28 °C under 150 rpm overnight. Following this, inoculums were transferred to 200 mL of the respective broth and cultured under the same conditions until an optical density corresponding to 10^8^ CFU mL^−1^ was reached, as described in previous studies [9,23].

A treatment and a control group were used for each bacterium. The water temperature was maintained at 28 °C for the *S. agalactiae* SA95-infected group (GSA) and its control group (GCSA), while for the *F. orientalis* FNO12-infected group (GFO) and its control group (GCFO), the water temperature was maintained at 22 °C. Six tambaqui juveniles were used per group, as summarized in Table 1. The number of fish per group was determined using the sample size formula for dichotomous data recommended by Dell et al. [28].

The experimental infection lasted for a period of 15 days, starting on the day of infection. Fish were fasted for 24 h before infection. Immediately before the infection procedure, the fish were anesthetized by immersion in benzocaine solution (Sigma-Aldrich; 100 mg L^−1^) to minimize stress and discomfort.

For the infection trials, group GSA was intraperitoneally injected with 0.1 mL of BHI broth containing 1 × 10^8^ CFU mL^−1^ of *S. agalactiae* SA95. The control group for this trial, group GCSA, received 0.1 mL of sterile BHI broth via intraperitoneal injection. Similarly, group GFO was intraperitoneally injected with 0.1 mL of MHB containing 3.4 × 10^8^ CFU mL^−1^ of *F. orientalis* FNO12. The control group for this trial, group GCFO, received 0.1 mL of sterile MHB via intraperitoneal injection.

Throughout the infection period, the fish were maintained under the same conditions described for the acclimation period. The water temperature was set to 28 °C for the groups involved in the *S. agalactiae* trial (GSA and GCSA), and 22 °C for the groups involved in the *F. orientalis* trial (GFO and GCFO). During the infection period, clinical signs and mortality were meticulously recorded to monitor the progression of the infection.

Fish that died during the challenge period were subject to bacteriological and histopathological analysis to determine the bacteria associated with the death and characterize the tissue lesions. Brain and kidney samples from GSA and GCSA fish were streaked onto TSA and incubated at 28 °C for 48 h. Spleen and kidney samples from GFO and GCFO fish were streaked onto CHA and incubated at 28 °C for 96 h. Rapid identification using MALDI-TOF MS (matrix-assisted laser desorption/ionization time-of-flight mass spectrometry) was performed upon detection of bacterial colony. Brain, kidney, liver, and spleen were also collected for histopathological examination to assess tissue damage and inflammatory responses. At the end of the challenge period, surviving fish were euthanized in benzocaine bath (Sigma-Aldrich; 300 mg L^−1^), necropsied, and subjected to the same bacteriological and histopathological examinations as previously described.

### 2.3. Bacterial Identification by MALDI-TOF MS

MALDI-TOF was used to identify the reisolated bacteria. Procedures followed those described by Assis et al. [22]. A score of 2000 or higher ascertained bacteria species. Scores between 1999 and 1700 would indicate reliable identification at genus level. Scores below 1700 would indicate not reliable identification.

### 2.4. Histological Examination

Histopathological tests were conducted to assess tissue damage caused by *S. agalactiae* SA95 and *F. orientalis* FNO12 in *C. macropomum*. Fragments of brain, liver, posterior kidney, heart, and spleen were obtained from each fish following the infection period [29] and fixed in neutral buffered formalin for 24 h to preserve the tissue structure and prevent degradation [30].

After fixation, the organ samples underwent a dehydration process using ascending concentrations of ethanol, ranging from 70% to 100% (Êxodo científica, São Paulo, Brazil). This step was essential to remove water from the tissues, allowing them to be embedded in paraffin wax. The dehydrated samples were then clarified with xylene (Dinâmica, São Paulo, Brazil), to make the tissues transparent, and finally embedded in paraffin wax (Synth, São Paulo, Brazil).

Once the tissues were embedded in paraffin wax, thin sections (4 µm thick) were cut using a Leica RM2245 semi-automated rotary microtome (Leica Biosystems, Wetzlar, Germany). These thin sections were then mounted on glass slides and stained with hematoxylin–eosin (HE) and Gram stain [31]. HE staining provided a detailed view of the tissue architecture and cellular components, while Gram staining was intended for the identification of Gram-positive and Gram-negative bacteria within the tissues. The sections were examined under a Leica DM4000 B microscope (Leica Biosystems and documented using a Leica DFC 500 digital camera (Leica Biosystems).

## 3. Results

### 3.1. Streptococcus agalactiae Experimental Infection

Following the experimental infection with *S. agalactiae*, the tambaqui juveniles exhibited clinical signs that included lethargy and lack of appetite, which persisted until the second day post-infection (dpi). Notably, a high mortality rate of 83.4% (five out of six fish) was observed on the same day. Interestingly, clinical signs indicating nervous system involvement, such as erratic swimming or loss of equilibrium, were not observed prior to the death of the infected fish.

The surviving fish showed anorexia until the third dpi but began feeding on the fourth dpi and survived throughout the infection period. Bacteriological analysis revealed that *S. agalactiae* was successfully isolated from the brain and kidney of the deceased fish, confirming the presence of the pathogen in these organs (Table 2). Additionally, *Plesiomonas shigelloides* was isolated from the brain of one fish and from the kidneys of three fish, while *Aeromonas* sp. was isolated from the brain of one fish. However, these isolates were considered contaminants due to the growth of single colonies, indicating that they were not the primary cause of the observed mortality.

In contrast, the control group (GCSA) displayed only brief anorexia on the first day post-inoculation, with no additional clinical signs, mortality, or bacterial isolation during experimental period.

**Figure 1:**
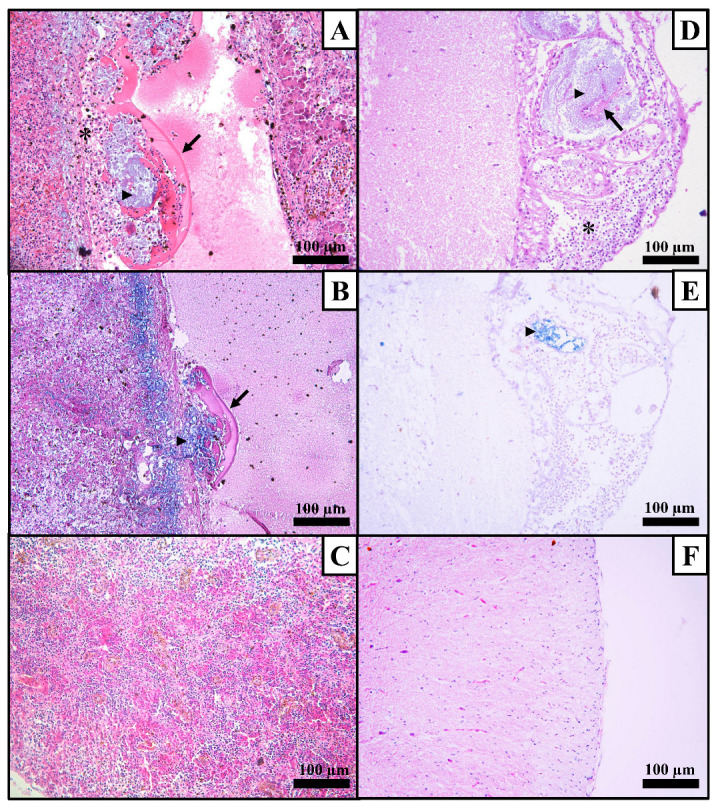
Histological images of organs from tambaqui (*Colossoma macropomum*) experimentally infected by *Streptococcus agalactiae* (SA95). (**A**) Fibrin thrombus (arrow) within a blood vessel of spleen constituted by aggregates of basophilic coccoid bacteria (arrowhead), surrounded with inflammatory infiltrate of neutrophils (asterisk) and accumulation of amorphous eosinophilic material (fibrin). H&E stain, 20× magnification. (**B**) Gram-stained section of the previously referred thrombus (arrow) in the spleen evidencing the Gram-positive aggregates of coccoid bacteria (arrowhead) in the center of the lesion. Gram stain, 20× magnification. (**C**) Section of a non-infected spleen. H&E stain, 20× magnification. (**D**) Aggregates of basophilic coccoid bacteria (arrowhead) and fibrin (arrow) in the meninge’s blood vessels and intense mononuclear inflammatory infiltrate (asterisk) in the meninge of the brain. H&E stain, 20× magnification. (**E**) Gram-stained section of the previously referred lesion of the brain evidencing that the bacterial aggregates are Gram-positive cocci, stained in blue (arrowhead). Gram stain, 20× magnification. (**F**) Section of a non-infected brain. H&E stain, 20× magnification.

Macroscopic examination of the infected fish revealed hyperemia, characterized by visible reddening and inflammation in the liver and kidney. Histological analysis further revealed neutrophilic and fibrinonecrotic inflammation, along with bacterial aggregates in the spleen (five out of six fish), liver (three out of six fish), brain (two out of six fish), and heart (three out of six fish). The spleen was identified as the most severely affected organ, where large bacterial clusters surrounded by fibrin and cellular debris were found, indicating a strong inflammatory reaction and significant tissue damage (Figure 1).

### 3.2. Francisella orientalis Experimental Infection

Tambaqui infected with *F. orientalis* exhibited persistent anorexia throughout the experimental period. Despite clinical signs, no mortality was recorded in the challenged fish. Bacteriological analysis revealed that *F. orientalis* was successfully reisolated from all challenged fish, with varying frequencies of isolation from different organs. Specifically, the bacterium was reisolated from the spleen in 83.3% of the fish and from the kidney in 50% of the fish, as detailed in Table 2. In contrast, all fish from the control group (GCFO) survived until the end of the experimental trial, exhibiting only brief anorexia on the first day following inoculation, with no additional clinical signs.

**Figure 2:**
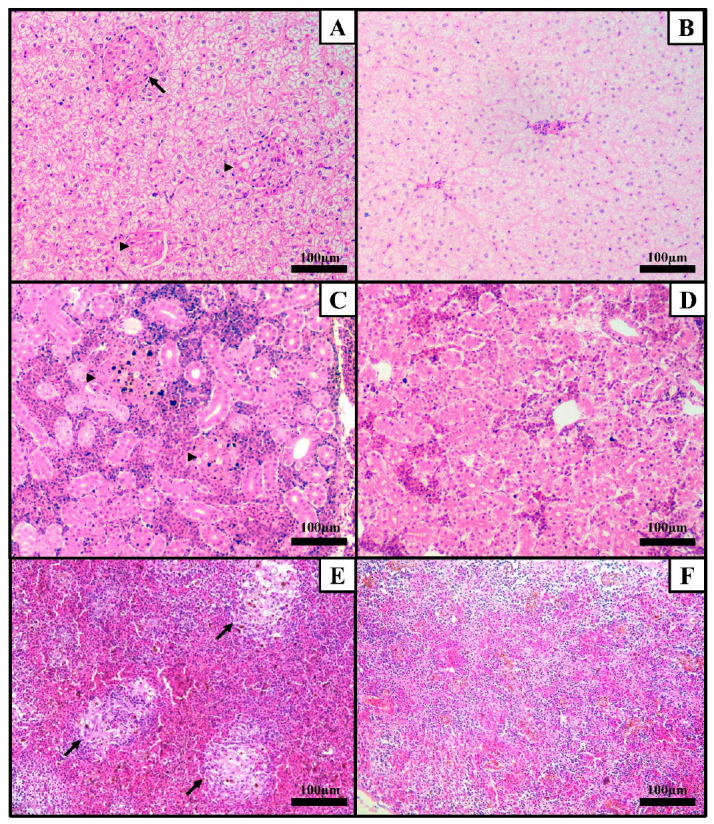
Histological images of organs from tambaqui (*Colossoma macropomum*) experimentally infected by *Francisella orientalis* (FNO12). (**A**) Large mature granuloma (arrow) surrounded by a thin wall of flattened macrophages and two immature granulomas (arrowhead) in the liver. All granulomas are predominantly constituted of macrophages and epithelioid macrophages. (**B**) Section of a non-infected liver. (**C**) Section of caudal kidney with increased cellularity between the tubules constituted of mononuclear inflammatory infiltrate and multifocal areas of immature granulomas (arrowhead) predominantly composed of macrophages and epithelioid macrophages and rare lymphocytes. (**D**) Section of a non-infected caudal kidney. (**E**) Large mature granulomas (arrows) in spleen. Granulomas are predominantly constituted of macrophages, epithelioid macrophages, rare lymphocytes, and pigmented macrophages and surrounded by a thin wall of flattened macrophages. (**F**) Section of a non-infected spleen. All sections were H&E stained. Magnification of 20×.

Histological examination of tambaqui juveniles inoculated with *F. orientalis* revealed granulomatous inflammation in the kidney (two fish affected), spleen (two fish affected), and liver (five fish affected) (Table 2), although the granulomas lacked necrotic centers (Figure 2). Interestingly, Gram staining failed to detect the Gram-negative nature of the causative bacteria.

## 4. Discussion

In this study, we investigated the potential pathogenicity of *S. agalactiae* and *F. orientalis*, previously isolated from diseased Nile tilapia, to tambaqui (*C. macropomum*). Our experimental infection trials in juvenile tambaqui, conducted at a dose of approximately 10^7^ CFU fish^−1^, revealed distinct differences in the pathogenicity of these two bacteria. *S. agalactiae* exhibited high pathogenicity, causing rapid disease progression, high mortality (83.33%) within 48 h post-infection, and severe lesions in multiple organs. In contrast, *F. orientalis* infection did not result in mortality or clinical signs, although bacterial recovery and granulomatous inflammation were observed in the tissues.

Regarding the *S. agalactiae* infection trial, *S. agalactiae* causing high mortality rates such as observed in tambaqui (83.3%) has been reported in other fish species. Tilapia infected intraperitoneally with approximately 1 × 10^7^ CFU/fish of *S. agalactiae* resulted in mortality rates ranging from 90% to 100% with most deaths occurring within three dpi [9,32]. In tilapia, massive proliferation and severe progression of lesions appears to be a common strategy for *S. agalactiae* infections [33], which was also observed in tambaqui. The median lethal dose of SA95 in tilapia was previously determined as 2.4 × 10^4^ CFU with common streptococcosis clinical signs observed at this dose [34]. For tambaqui, a median lethal dose is yet to be established. Nevertheless, no mortality was reported after tambaqui infection with 10^5^ CFU of *S. agalactiae*/fish [35]. This could be attributed to the use of a strain isolated from a healthy human (*Streptococcus agalactiae* ATCC 13813). On the other hand, strain SA95 belongs to a well-adapted genetic lineage of *S. agalactiae* (clonal complex CC 260) capable of infecting tilapia [34], which explains the pathogenicity observed. Nevertheless, the quick progression of disease observed in tambaqui is most likely also related to the dose employed.

Despite the rapid disease progression, tissue lesions were still observed, revealing the occurrence of neutrophilic and fibrinonecrotic inflammation in the spleen, brain, liver, and heart. These findings are consistent with those reported in other fish hosts, where similar inflammatory responses have been documented [32,33,36,37,38]. Neutrophilic inflammation, characterized by the infiltration of neutrophils, is a common immune response to bacterial infections and indicates an acute inflammatory reaction [39]. Fibrinonecrotic inflammation, on the other hand, involves the deposition of fibrin and necrosis of tissue, reflecting severe tissue damage and a more advanced stage of infection [40]. The presence of these lesions in multiple organs highlights the systemic nature of the infection and the significant impact it has on the health of the affected fish.

The bacteriological analysis revealed the presence of *Plesiomonas shigelloides* and *Aeromonas* sp. in some fish challenged with *S. agalactiae* SA95. These bacteria are commonly found in freshwater environments and, while they possess pathogenic potential to fish, their isolation from infected tambaqui is likely attributed to post mortem invasion [41,42] or even a secondary infection in SA95-infected animals [43], since no contaminants were found in the control group.

*S. agalactiae* serotype Ib is a pathogen predominantly associated with humans and fish [44,45]. Although *O. niloticus* is the most affected fish, other species such as *Puntius conchonius, Mikrogeophagus ramirezi* [46], *Epinephelus lanceolatus, Pomadasys kaakan, Arius thalassinus, Liza vaigiensis, Aptychotrema rostrata, Himantura granulate*, and *Dasyatis fluviorum* have also been reported as vulnerable hosts [47]. In Brazil, the hybrid *Leiarius marmoratus × Pseudoplatystoma corruscans* (Amazon catfish) has also been affected by *S. agalactiae* serotype Ib [48]. The strain found affecting the Amazon catfish is part of the ST-260 (a single locus variant of ST-927 found in SA95) which is the main ST isolated from tilapia in Brazil, demonstrating the adaptability of this pathogen to hosts. Time-measured analyses such as Bayesian evolutionary phylogeny indicate that ST-927 (the sequence type of SA95 strain) diverged from other piscine *S. agalactiae* STs within Brazil only 46 years ago, and other STs have emerged since then, which evidences how quickly this microorganism adapts [21]. Tambaqui were shown to be susceptible to *S. agalactiae* Ib in this study, suggesting the potential for this pathogen to occur or to adapt naturally in this target-host.

*S. agalactiae* that affect Brazilian tilapia are commonly serotyped as Ib, but serotypes III and Ia also occur [21,48,49]. The data shown in this study are restricted to *S. agalactiae* serotype Ib, and further analyses are needed to determine how other serotypes interact with tambaqui. This should address the risks that other serotypes may pose to tambaqui production.

*F. orientalis* is a Gram-negative intracellular pathogen that mainly invades macrophages once inside its host. This feature allows *F. orientalis* to effectively evade the immune system, extending its survival. Although *F. orientalis* is mainly reported in tilapia, the vulnerability of the following fish species has already been reported to this pathogen: *Haemulon flavolineatum*, *Haemulon carbonarium*, *Haemulon aurolineatum*, *Anisotremus virginicus*, *Haemulon sciurus*, *Haemulon chrysargyreum*, *Haemulon parra*, *Haemulon macrostomum* [50], *Cirrhilabrus* spp., *Chromis viridis* [51], *Hemichromis bimaculatus* [52], and *Herichthys cyanoguttatus* [53]. The host diversity is limited compared to *S. agalactiae*. This may be explained by how clonal *F. orientalis* populations are, including FNO-12 [24,54,55], which limits the ability of this pathogen to naturally infect new hosts. Still, under the experimental conditions, *F. orientalis* was proved to trigger pathogenic effects in tambaqui.

Granulomatous inflammation, a common manifestation of the pathogen in other fish species [56] was also observed in infected tambaqui. Notably, common carp (*Cyprinus carpio*) and panga (*Pangasianodon hypophthalmus*) experimentally infected with *F. orientalis* did not exhibit clinical signs or mortality. The bacterium was not reisolated and did not induce histopathological changes, suggesting a lack of pathogenicity in these hosts [57]. Similarly to *F. orientalis*, *E. tarda* did not induce mortality or clinical signs in tambaqui, whilst bacterial recovery and granulomas on histological examination were present [8]. Reisolation of *F. orientalis* occurred from all infected tambaqui juveniles; however, reisolation from spleen (83.3%) was considerably higher than reisolation from posterior kidney (50%). In tilapia infected by *F. orientalis*, spleen is frequently reported as the most affected organ, which is most likely associated with higher macrophage density observed in this organ [20,58] compared to posterior kidney, which is also a renal organ [59]. While Gram stain failed to highlight bacterial cells, the validation of recovered bacteria identity and the typical histological findings suggest that the lesions were indeed induced by *F. orientalis*. In contrast to the lack of mortality in tambaqui, common carp, and panga, *F. orientalis* has been reported to cause mortality rates of 100%, 64.28%, and 21.42% in Nile tilapia, giant perch (*Lates calcarifer*), and largemouth bass (*Micropterus salmoides*), respectively, with reisolation rates of 82.71%, 35.71%, and 21.42% [60]. Characteristic clinical signs and histopathological alterations were also noted. Granulomas in Nile tilapia surge as early as three dpi by *F. orientalis* in organs such as spleen and anterior kidney [61]. In tambaqui, the time to form the first granulomas could not be addressed since fish were examined only at the end of the infection period. Granulomas are an immune system reaction aimed at isolating the foreign material [62]. However, for intracellular microorganisms, they may in fact promote the spread of the pathogen by gathering macrophages that will be invaded by *F. orientalis,* similarly to what was described in *Mycobacterium marinum* infections [63]. Ultimately, the fast granuloma formation in Nile tilapia progresses to forming macroscopic white nodules. In tambaqui, these macroscopic lesions were not observed, which may indicate that *F. orientalis* is more pathogenic to Nile tilapia. Posterior studies should aim at assessing the chronicity of *F. orientalis* infections in *C. macropomum*. Overall, these findings indicate that the pathogenicity of *F. orientalis* varies significantly across different fish species.

In our study, tambaqui infected with *F. orientalis* were maintained at 22 °C, as lower temperatures appear to enhance the pathogenicity of this bacterium in tilapia, with a reduced median lethal dose observed in tilapia kept at 22 °C (2.4 × 10^2.21^ CFU) compared to 28 °C (1.64 × 10^5.74^ CFU) [23]. However, *F. orientalis* demonstrates adaptability to both 22 °C and 28 °C by modifying the expression of genes related to metabolism, oxidative stress, and heat shock response [23]. Moreover, the pathogenicity island genes of *F. orientalis* are highly expressed at both temperatures, suggesting a strong virulence potential across the temperature range [23]. Furthermore, *F. orientalis* has been shown to induce disease and lethality in zebrafish maintained at 28 °C [64]. Therefore, subsequent studies are necessary to evaluate the pathogenicity of *F. orientalis* in tambaqui at 28 °C, a temperature frequently encountered in the farming of tambaqui, as its virulence genes are highly expressed at both 22 °C and 28 °C.

A limitation of this study is that horizontal transmission from tilapia to tambaqui was not investigated. It is of utmost importance to address this issue, but experimental designs employing cohabitation and immersion infection models to assess whether these pathogens trespass the physical and immunological barriers of tambaqui will be required. For instance, *S. agalactiae* Ib and *F. orientalis* adhesion and invasion through intestines were already demonstrated in tilapia but not yet in tambaqui [33,61,65]. These questions remain unanswered and should be explored in future research.

## 5. Conclusions

Our findings indicate that *S. agalactiae* serotype Ib isolated from tilapia is highly pathogenic to tambaqui, inducing rapid disease progression and high mortality under the experimental conditions. Conversely, *F. orientalis*, also isolated from tilapia, was able to infect and persist in the host, but did not cause mortality or significant clinical signs under the experimental conditions. Although these pathogens were not isolated from tambaqui, their potential to infect this species and cause disease should be considered in tambaqui farming. The simultaneous cultivation of both fish species or even cultivation of only one species while the other is present in the same environment may pose a great risk of cross-species barrier transmission events.

## Figures and Tables

**Table 1:** Experimental infection design.

Group	Inoculum	Water Temperature	No. of Fish
GSA	SA95 strain (1 × 10^7^ CFU fish^−1^) + BHI broth	28 °C	6
GCSA	Sterile BHI broth	28 °C	6
GFO	FNO12 strain (3.4 × 10^7^ CFU fish^−1^) + MHB	22 °C	6
GCFO	Sterile MHB	22 °C	6

**Table 2:** Mortality, bacterial reisolation, and histology lesions following experimental infection of *Colossoma macropomum* with *Streptococcus agalactiae* and *Francisella orientalis*.

Bacteria	Fish	Death	Bacterial Reisolation			Histological Alterations			
			Brain	Kidney	Spleen	Brain	Kidney	Liver	Spleen
*Streptococcus* *agalactiae*	1	Yes	+*	+*	NA	−	−	+	+
	2	Yes	+*	+*	NA	+	−	−	+
	3	Yes	+	+*	NA	−	+	+	+
	4	Yes	+	+	NA	+	−	+	+
	5	Yes	+	+	NA	−	−	+	+
	6	No	−	−	NA	NA	NA	NA	NA
	Total	5/6	5/6	5/6	NA	2/5	1/5	4/5	5/5
*Francisella* *orientalis*	1	No	NA	−	+	−	−	−	+
	2	No	NA	+	+	−	−	+	−
	3	No	NA	−	+	−	+	+	−
	4	No	NA	+	+	−	−	+	+
	5	No	NA	+	−	−	−	+	−
	6	No	NA	−	+	−	+	+	−
	Total	0/6	NA	3/6	5/6	0/6	2/6	5/6	2/6

(+) Bacteria or histology alterations are present; (−) bacteria or histology alterations are absent; (+*) *S. agalactiae* was reisolated and another microorganism was also present as a single colony; (NA) not analyzed.

## Data Availability

The original contributions presented in the study are included in the article, further inquiries can be directed to the corresponding author.
